# Effect of exogenous surfactants on viability and DNA synthesis in A549, immortalized mouse type II and isolated rat alveolar type II cells

**DOI:** 10.1186/1471-2466-11-11

**Published:** 2011-02-17

**Authors:** Andreas Wemhöner, Paul Jennings, Thomas Haller, Mario Rüdiger, Georg Simbruner

**Affiliations:** 1 Innsbruck Medical University, Department for Pediatrics, Neonatology; Austria; 2 Technical University Dresden, University Hospital Dresden, Department for Pediatric Intensive Care and Neonatology; Germany; 3 Innsbruck Medical University, Department of Physiology and Medical Physics; Austria

## Abstract

**Background:**

In mechanically ventilated preterm infants with respiratory distress syndrome (RDS), exogenous surfactant application has been demonstrated both to decrease DNA-synthesis but also and paradoxically to increase epithelial cell proliferation. However, the effect of exogenous surfactant has not been studied directly on alveolar type II cells (ATII cells), a key cell type responsible for alveolar function and repair.

**Objective:**

The aim of this study was to investigate the effects of two commercially available surfactant preparations on ATII cell viability and DNA synthesis.

**Methods:**

Curosurf^® ^and Alveofact^® ^were applied to two ATII cell lines (human A549 and mouse iMATII cells) and to primary rat ATII cells for periods of up to 24 h. Cell viability was measured using the redox indicator resazurin and DNA synthesis was measured using BrdU incorporation.

**Results:**

Curosurf^® ^resulted in slightly decreased cell viability in all cell culture models. However, DNA synthesis was increased in A549 and rat ATII cells but decreased in iMATII cells. Alveofact^® ^exhibited the opposite effects on A549 cells and had very mild effects on the other two cell models.

**Conclusion:**

This study showed that commercially available exogenous surfactants used to treat preterm infants with RDS can have profound effects on cell viability and DNA synthesis.

## Background

Respiratory Distress Syndrome (RDS) is caused by surfactant deficiency and is a leading cause of mortality and morbidity in preterm newborns. Some infants develop the chronic lung disease, bronchopulmonary dysplasia (BPD), which is a major cause of long term morbidity [1]. BPD, as described by Northway [2], is mainly caused by a pulmonary injury associated with mechanical ventilation [3]. The subsequent histological correlate is characterised by cellular proliferation and fibrosis at the alveolar level [4,5]. Another entity of chronic lung disease is "New BPD" which occurs even without any mechanical ventilation. New BPD is characterized by abnormal development of alveolar and capillary structures [5].

A key finding in both forms of chronic lung disease is an abnormal cellular proliferation in cells of the alveolus. Type II pneumocytes (ATII cells) are one of the major alveolar cell types and function to synthesise, secrete and recycle surfactant, and are also involved in immunological processes. In response to lung injury, ATII cells proliferate and differentiate into type I cells to maintain the alveolar structure and function [6]. However, it is still poorly understood which factors affect their differentiation and proliferation [7].

Administration of exogenous surfactant represents the established therapy of RDS. Exogenous surfactant does not only reduce surface tension, but has also been shown to suppress proliferation of lymphocytes in a concentration dependent manner [8]. In isolated fibroblasts, surfactant causes a decrease of DNA-synthesis [9]. Observations in autopsies from infants with BPD suggested a possible acceleration of epithelial cell regeneration in those receiving surfactant [10]. Another study found histological evidence of ATII cell hyperplasia and dysplasia in infants treated with surfactant [11]. In contrast, a third study did not find differences with respect to cellular proliferation and metaplasia between surfactant-treated and control infants [12].

In summary, the information on the effect of surfactant on ATII cell proliferation is inconsistent and has not been studied on the cellular level to date. Therefore, the present study was performed in cultured ATII cells to investigate potential mitogenic or toxic effects of two commercial surfactant preparations.

## Methods

### Materials

All materials were of the highest analytical grade and were purchased from Sigma, Vienna, Austria, unless otherwise stated. Curosurf^® ^(Nycomed, Vienna, Austria) is produced from minced porcine lungs and Alveofact^® ^(Boehringer-Ingelheim, Biberach, Germany) is obtained from bovine lung lavage. Both preparations contain surfactant associated proteins B and C, however, at different concentrations. Furthermore, the lipid composition and concentration differ between both preparations [13].

### Cell cultures

A549 cells: This human epithelial type II-like cell line was cultured in Dulbecco's modified Eagle's medium (DMEM) supplemented with 7% foetal calf serum (FCS), 100 U/ml penicillin G and 100 μg/ml streptomycin at 37°C. A549 cells were obtained from ATCC (American Tissue Culture Collection, USA).

Mouse cells: The immortal mouse alveolar type II cells (iMATII) were obtained from the original developing laboratory [14]. iMATII cells were seeded on human collagen IV (5 μg/cm²) coated cell culture dishes (density of 5 × 10^5 ^cells/ml) in DMEM with 5 mM glucose, supplemented with 7% FCS, 10 ng/ml human Epithelial Growth Factor (EGF) and 10 ng/ml mouse interferon gamma. Cells were incubated at 33°C in DMEM medium (100 U/ml penicillin G and 100 μg/ml streptomycin, 10 nM dexamethasone, 0.1 mM 8-bromoadenosine 3',5'-cyclic monophosphate cAMP, and 0.1 mM isobutylmethylxanthine [15].

Rat cells: Rat ATII (RTII) cells were isolated from male Sprague-Dawley rats (200 g), according to the procedure of Dobbs et al. [16] with slight modifications as described by Haller et al. [17]. Animal care and use were approved by the Institutional Animal Care and Use Committee of the Innsbruck Medical University (ZI A 07/3456). In short, lungs were cleared of blood by perfusion and removed from the thorax. After lavage, the lungs were instilled with elastase solution (30 units/ml), incubated at 37°C, and minced in the presence of DNase. After stopping the elastase reaction by addition of FCS, the cell suspension was sequentially filtered and centrifuged. The cell pellet was re-suspended in DMEM and panned on IgG-coated plastic dishes at 37°C to remove macrophages. Cells were centrifuged, suspended in DMEM supplemented with 10% FCS, 100 units/ml penicillin, 100 μg/ml streptomycin, and 24 mM NaHCO_3 _and cultured in 95% humidified air and 5% CO_2 _at 37°C.

After cell preparation, cells were plated at a density of 4 × 10^4 ^cells/ml in 96 well plates (Sarstedt, Nümbrecht, Germany) and were incubated at 37°C.

### Surfactant incubation

**Figure 1:**
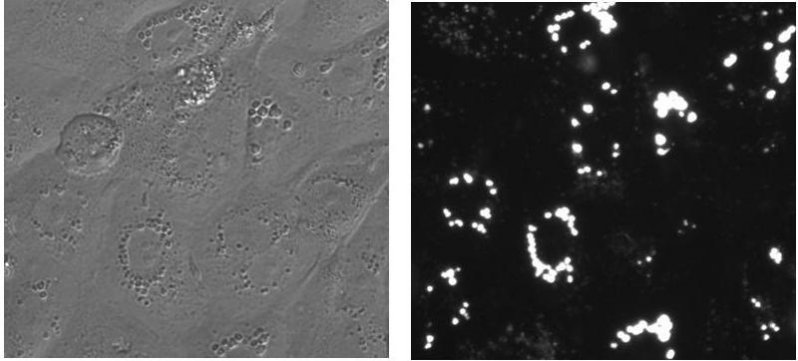
**Rat ATII cells imaged by phase contrast (left) and fluorescence (480 nm), 24 h after incubation with Curosurf^®^**. Intracellular Lamellar Bodies, the characteristic surfactant storing organelles, were stained with 500 nM LysoTracker Green DND-26. Noticeable morphological differences to control cells were not detected.

After 24 h, cells were washed twice with DMEM to remove non-adherent cells. Confluency was in the range of 60-70%. Thereafter, surfactant was added for the long term incubation (24 h; Figure 1). Short term incubations (0.5 and 4 h) were started after 23.5 or 20 h, respectively. Curosurf^® ^or Alveofact^® ^was diluted with DMEM to achieve a final concentration of 10 mg phospholipids per ml [18]. At treatment time, medium was removed from the wells and replaced by 150 μl of the diluted surfactants. For controls, cells were incubated with fresh DMEM alone.

### Determination of cell viability

The amount of viable cells was measured by tracing the metabolic conversion of resazurin to resorufin. Cells were incubated with 150 μl DMEM containing 44 μM resazurin for 60 min as previously described [19] Viable cells convert resazurin to the fluorescent metabolite resorufin, which was measured at 540 nm excitation and 590 nm emission using a fluorescence plate reader (GENios Plus, Tecan, Austria).

### Determination of DNA synthesis

For measurement of DNA synthesis, a commercially available BrdU assay (Roche, Mannheim, Germany) was used as previously described [19]. The assay is based on BrdU incorporation into the DNA of proliferating cells.

### Statistics

Experiments were performed in triplicate with each cell type and treatment; each experiment had eight technical replicates. Differences between each surfactant preparation and controls were analyzed with the unpaired Student's t-test. A p < 0.05 vs. control was considered as statistically significant. The results are expressed as percentage of the value of DMEM-incubated control cells of the same experiment. All data are represented as arithmetic means + standard errors of the means (SEM).

## Results

### Effects of incubation time

**Figure 2:**
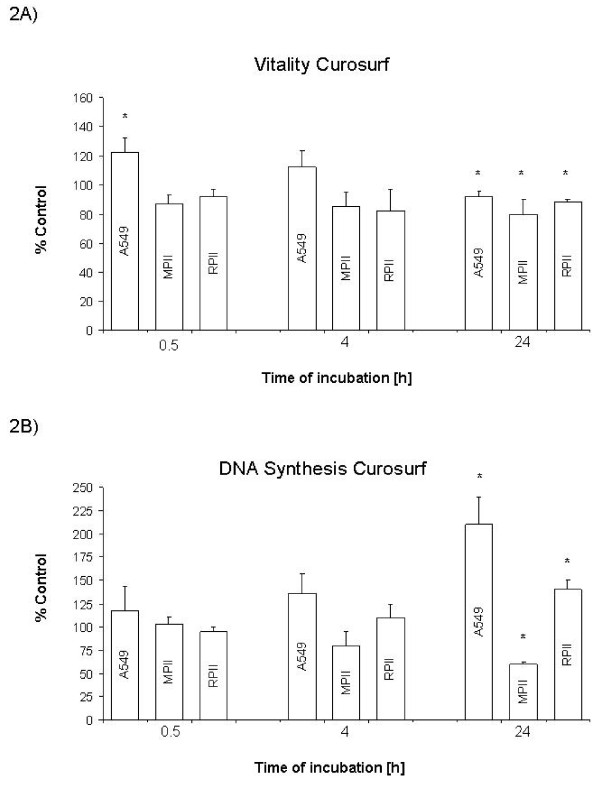
**Effect of Curosurf^® ^on viability and DNA synthesis**. Cells were exposed to 10 mg/ml Curosurf^® ^for 0.5, 4.0 and 24 h as described. A: Viability as percentage of control. B: DNA synthesis as percentage of control. Bars represent mean + SEM of 3 independent experiments. * *P *<0.05 vs. control. MPII: mouse type II (iMATII) cells, RPII: rat type II cells.

**Figure 3:**
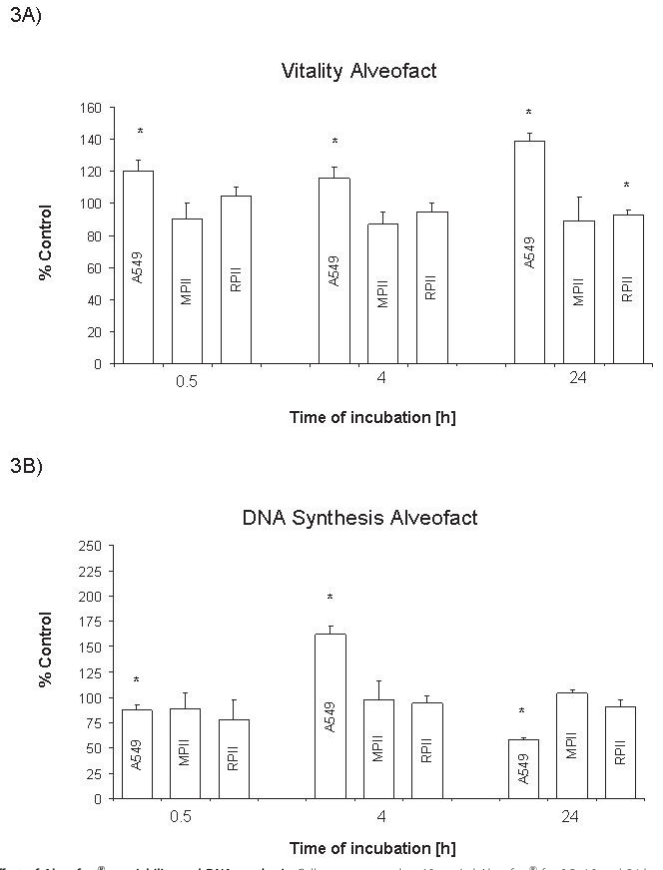
**Effect of Alveofact^® ^on viability and DNA synthesis**. Cells were exposed to 10 mg/ml Alveofact^® ^for 0.5, 4.0 and 24 h as described. A: Viability as percentage of control. B: DNA synthesis as percentage of control. Bars represent mean + SEM of 3 independent experiments. * *P*<0.05 vs. control. MPII: mouse type II (iMATII) cells, RPII: rat type II cells.

Curosurf^® ^exposure resulted in a time dependent decrease in cell viability in all cell models (Figure 2A) and a time dependent increase in proliferation in A549 and RPII cells, but a time dependent decrease in iMATII cells (Figure 2B**)**. Alveofact^® ^exposure had no apparent temporal effect in cell viability in any of the cell culture models tested (Figure 3, A and 3B). However, Alveofact^® ^exposure resulted in a stimulation of DNA synthesis at 4 h in A549 cells, which was significantly inhibited at 24 h. Viability of control cells increased in all cell models after 24 hours, but not after 4 hours (Table 1.).

**Table 1:** Viability and DNA synthesis of control cells

	4 hours			24 hours		
	A549	MPII	RPII	A549	MPII	RPII

Viability	104 ± 4	105 ± 5	106 ± 4	122 ± 8*	117 ± 6*	108 ± 3*

DNA synthesis	100 ± 3	103 ± 3	100 ± 11	115 ± 5*	122 ± 8*	103 ± 3

*P < 0.01 vs. 100% (= mean RFU values at 0.5 hours) MPII: mouse type II (iMATII) cells, RPII: rat type II cells

### Effect of surfactant on different cell models

Cell model differences were evident with both surfactant preparations. Curosurf^® ^had opposite effects on DNA synthesis, stimulating A549 and RPII cells, while inhibiting iMATII cells (Figure 2B). Alveofact^® ^exposure showed comparable results with the iMATII and RPII cells, having little effect on cell viability and no effect on DNA synthesis, whereas in A549 cells, Alveofact^® ^caused an apparent increase in cell viability and time-dependently affected DNA synthesis (Figure 3A and 3B). DNA synthesis of control cells increased in A549 and MPII cells after 24 hours (Tab. 1).

## Discussion

Preterm infants with RDS are treated with exogenous surfactant. Surfactant has been shown to affect proliferation and DNA synthesis in fibroblasts and lymphocytes [8,9]. Furthermore, surfactant treatment may accelerate epithelial cell regeneration in mechanically ventilated preterm infants with RDS [10]. The present study investigated the effect of exogenous surfactant on DNA synthesis of isolated rat ATII cells and ATII like cells (A549) and mouse type II cells (iMATII). We demonstrated that exogenous surfactant alters DNA synthesis and viability of cultured type II cells differentially. The effect of surfactant on both viability and DNA synthesis was dependent on the duration of contact, type of surfactant and species of origin of ATII cells.

Duration of surfactant incubation affected mainly incorporation of BrdU into the cells, a surrogate of DNA synthesis and cell proliferation, and to a lesser extent cellular viability. The mechanism of surfactant-mediated alterations in cellular viability and its relation to proliferation are complex, and a direct correlation between cell viability and DNA synthesis cannot be expected in all situations, also in control cells. The resazurin assay measures cellular redox potential, which, for the most part, is an excellent indicator of cell viability. However, exogenous stimuli may result in an increase in cellular redox potential which may also be considered as a perturbation of cellular function whilst not directly related to cell viability or number. This might explain the seemingly paradoxical situation of an increased DNA synthesis despite an increased apparent viability, particularly seen in the A549 cells. Alternatively, a decrease in cell viability may directly stimulate cellular proliferation as occurs in tissue repair. This effect is seen with Curosurf^® ^in A549 cells. Finally, cellular damage in the absence of repair will result in a direct correlation of these parameters, decreased cell number and decreased DNA synthesis, as might be the case in iMATII cells treated with Curosurf^®^.

Various commercially available surfactant preparations are used to treat RDS in preterm infants. Whereas surface tension lowering properties seem to be similar, other properties such as biochemical composition, morphological organisation and immunological functions differ. Native surfactants contain SP-A/B/C and SP-D, 1.5% SP-B and 2.8 to 4.5% SP-C per μmol phospholipids. Commercial surfactants contained only one half to one third of these proteins as compared with native surfactants and no SP-A and SP-D components [20].

Little is known about the effect on alveolar cells. In the present study, the effect of Curosurf^® ^and Alveofact^® ^was not the same. Surfactant lipids affect the fluidity of cellular membranes and could thus alter cellular pathways [9,21]. Higher concentrations of polyunsaturated fatty acid-containing phospholipids (PUFA-PL) were found in Curosurf^® ^compared with Alveofact^®^. They also have a difference in surface viscosity. Curosurf^®^, with the highest plasmalogen level and lowest cholesterol content has a viscosity of < 5 × 10^-6 ^kg/s compared to < 10 × 10^-6 ^kg/s of Alveofact^®^, which has a high concentration of cholesterol and small amounts of plasmalogens [13]. Consequently, surfactant preparations have been shown to have an inhibitory effect on lymphocyte proliferative responses to mutagens, alloantigen [22] and interleukin-2 [23,24]. Furthermore, surfactant affects immunoglobulin production by B cells and cytotoxicity of natural killer cells [22]. Surfactant interacts with macrophages [25] and inhibits cytokine release [21,26] down regulates DNA-synthesis of fibroblasts[9] and suppresses the lymphocyte proliferation in a concentration-dependent manner [8]. The latter effect was different when large surfactant aggregates were compared with small aggregates. While the former had minimal effects on DNA synthesis, the latter exhibited a bi-phasic response. The differences could be explained by differences in lipid composition of different surfactant subtypes [13,27].

Thus, it could be speculated, that differences found in the present study are due to differences in lipid composition of the surfactant preparations, however, subsequent studies are required to prove this hypothesis.

Comparisons of biological effects of exogenous surfactant in in-vitro models is often complicated by the fact that different cells types and species were utilized in each study.

Therefore, we carried out a single study with three ATII cell culture models in order to investigate whether or not such models are inherently different in their responses. As the results show, the effect of exogenous surfactant on viability or DNA synthesis can significantly vary depending on the ATII cell culture model used. A similar comparison has not been conducted previously. However, whether these differences are species related or cell model specific is difficult to clarify.

### Clinical relevance of the results

Clinical data of a retrospective study that compare infants treated with either Curosurf^® ^or Alveofact^® ^did not show any differences in the incidence of chronic lung disease [28]. Post mortem specimens of mechanically ventilated preterm infants with RDS who required supplemental oxygen and received exogenous surfactant showed no alterations in proliferation of epithelial cells when compared with lungs obtained from preterm infants who did not receive exogenous surfactant [29]. Other authors did not find a histological differences in lung specimens obtained from preterm infants who received surfactant or were untreated [12,30]. However, it is known, that surfactant treatment may accelerate epithelial cell regeneration in preterm infants with RDS significantly [31]. These seemingly contradictory results could be due to different surfactant preparations or different time points after surfactant therapies that were investigated. Whereas Gonda et al. focused on the early repair process in infants less than 7 days of age the other authors described long term effects. In conclusion, this study supports the hypothesis that exogenous surfactant can affect pulmonary alveolar cell DNA synthesis of extremely preterm infants, and that this effect is potentially dependent on the type of surfactant and on the duration of exposure.

## Competing interests

The authors declare that they have no competing interests.

## Authors' contributions

AW contributed to the conception and design of the study, calculated the data and wrote in part the manuscript. PJ contributed substantially to experimental design and methodological implementation and with TH aided in manuscript preparation. MR and GS were responsible for general interpretation of this study and drafting the manuscript. All authors read and approved the final manuscript.

## Pre-publication history

The pre-publication history for this paper can be accessed here:

http://www.biomedcentral.com/1471-2466/11/11/prepub
